# Complete Genome Sequence of *Vibrio coralliilyticus* Strain OCN014, Isolated from a Diseased Coral at Palmyra Atoll

**DOI:** 10.1128/genomeA.01318-14

**Published:** 2014-12-18

**Authors:** Blake Ushijima, Patrick Videau, Donna Poscablo, Veronica Vine, May Salcedo, Greta Aeby, Sean M. Callahan

**Affiliations:** a Department of Microbiology, University of Hawai‘i, Honolulu, Hawai‘i, USA; b Hawai‘i Institute of Marine Biology, Kāne‘ohe, Hawai‘i, USA

## Abstract

*Vibrio coralliilyticus* is a marine gammaproteobacterium that has been implicated as an etiological agent of disease for multiple coral genera on reefs worldwide. We report the complete genome of *V. coralliilyticus* strain OCN014, isolated from a diseased *Acropora cytherea* colony off the western reef terrace of Palmyra Atoll.

## GENOME ANNOUNCEMENT

*Vibrio coralliilyticus* is a marine bacterium that can cause disease in a range of coral species, including *Pocillopora damicornis* (1), *Pachyseris speciosa*, *Montipora aequituberculata*, *Acropora cytherea* (2), and *Montipora capitata* (3). *V. coralliilyticus* has also been shown to infect fish (4) and multiple bivalve genera (5, 6). Strains of *V. coralliilyticus* have been isolated from diseased corals in the Indian (1), Pacific (2), and Atlantic oceans (7). Coral disease caused by *V. coralliilyticus* is generally characterized by temperature-dependent infections resulting in bleaching or tissue lysis (8). In contrast, infections caused by the recently described *V. coralliilyticus* strain OCN008 were not stringently regulated by water temperature and did not cause bleaching (3). The success of this pathogen has been attributed to a vast repertoire of putative virulence factors proposed based on genomic, proteomic, and transcriptomic studies (3, 9, 10).

*V. coralliilyticus* strain OCN014 was isolated from a colony of *Acropora cytherea* exhibiting disease signs of *Acropora* white syndrome (AWS) off a reef at Palmyra Atoll. AWS was first observed at Palmyra Atoll in 2010 after a mild bleaching event in 2009 (11). A fragment of diseased *A. cytherea* was crushed and plated on thiosulfate citrate bile salts sucrose (TCBS) agar (Sigma-Aldrich) as previously described (12). Genomic DNA was isolated as previously described (13) and sequenced using the Roche 454 GS FLX Titanium system and Ion Personal Genome Machine sequencer technology at the Advanced Studies of Genomics, Proteomics, and Bioinformatics Core Facility (Honolulu, HI, USA; http://www.asgpb.mhpcc.hawaii.edu). The high-throughput sequencing reads yielded 887,469,519 bp of sequence data, and the 4,605,000 reads were assembled using the Newbler version 2.8 software into 42 contigs, with an average contig size of 135 kb. Gaps were filled using PCR and subsequent Sanger sequencing at the Pacific Biosciences Research Consortium Biotech Core Facility (Honolulu, HI; http://core.biotech.hawaii.edu). Annotation was conducted using the NCBI Prokaryotic Genome Annotation Pipeline. General analysis was conducted using the Rapid Annotation using Subsystem Technology (RAST) server (14).

The complete OCN014 genome consists of 5,732,794 bp with an average GC content of 45.7%. The genome is contained on two large chromosomes, chromosome 1 and 2, and a plasmid, pOCN014, which are 3,463,115 bp, 1,888,898 bp, and 380,781 bp in size, respectively. A total of 19 rRNA and 86 tRNA coding sequences were annotated. To our knowledge, this is the first complete *V. coralliilyticus* genome sequence published that contains no gaps in sequence data.

### Nucleotide sequence accession numbers.

This whole-genome shotgun project has been deposited at DDBJ/EMBL/GenBank under the accession numbers CP009264, CP009265, and CP009266, which refer to chromosome 1, chromosome 2, and pOCN014, respectively. The versions described in this paper are CP009264.2, CP009265.2, and CP009266.2. The BioProject ID is PRJNA258550.
